# Presence of EBV and HHV-7 Genomic Sequences in Saliva and Virus-Specific Antibodies in Sera of Oral Lichen Planus and Aphthous Stomatitis Patients: A Comparative Observational Study

**DOI:** 10.3390/v18050506

**Published:** 2026-04-28

**Authors:** Jagriti Kakar, Liba Sokolovska, Maksims Zolovs, Modra Murovska, Ingrīda Čēma

**Affiliations:** 1 Centre of Oral Medicine, Institute of Stomatology, Riga Stradiņš University, 20 Dzirciema Str., LV-1007 Riga, Latvia; ingrida.cema@rsu.lv; 2 Institute of Microbiology and Virology, Riga Stradiņš University, 5 Rātsupītes Str., LV-1067 Riga, Latvia; liba.sokolovska@rsu.lv (L.S.); modra.murovska@rsu.lv (M.M.); 3 Statistics Unit, Riga Stradiņš University, 16 Dzirciema Str., LV-1007 Riga, Latvia; maksims.zolovs@rsu.lv; 4 Institute of Life Sciences and Technology, Daugavpils University, 1A Parades Str., LV-5401 Daugavpils, Latvia

**Keywords:** Epstein–Barr virus, human herpesvirus 7, oral lichen planus, recurrent aphthous stomatitis, salivary diagnostics, viral load, real-time PCR, ELISA, oral mucosal inflammation, oral immunopathology

## Abstract

The presence of human herpesviruses is frequently detected in the oral cavity, yet their disease-specific role in chronic inflammatory oral mucosal disorders remains uncertain. This comparative observational study investigated Epstein–Barr virus (EBV) and human herpesvirus-7 (HHV-7) genomic sequences in saliva and virus-specific antibodies in serum among patients with oral lichen planus (OLP; n = 35), aphthous stomatitis (AS; n = 31), and healthy controls (n = 34). Salivary viral loads were quantified using real-time PCR, while EBV and HHV-7-specific IgG and IgM antibodies were measured using ELISA-based assays. EBV and HHV-7 DNA in saliva were commonly detected across all groups, demonstrating high baseline shedding and marked interindividual variability. Although EBV IgG levels were higher in OLP compared with AS in univariate analysis, multivariate regression revealed that age, rather than disease status, was the primary determinant of EBV IgG levels. After adjustment for age, sex, and discomfort, neither EBV nor HHV-7 salivary loads showed independent associations with OLP or AS. HHV-7 salivary loads were uniformly distributed among groups. These findings suggest that salivary detection of EBV and HHV-7 reflects widespread latent infection rather than disease-specific activity in OLP or AS. Longitudinal and tissue-based studies integrating immunological profiling are warranted to clarify whether herpesvirus reactivation contributes to disease severity in defined patient subgroups.

## 1. Introduction

Oral lichen planus (OLP) is a chronic immune-mediated disorder with recognized malignant transformation potential requiring long-term clinical monitoring [1], whereas recurrent aphthous stomatitis (RAS or AS) is a common inflammatory condition of the oral mucosa characterized by recurrent painful ulcerations of multifactorial etiology [2], which significantly impair patients’ quality of life. Their etiopathogenesis is considered multifactorial, involving genetic susceptibility, dysregulated cell-mediated immunity, psychological stress, and environmental influences [3,4]. Viral factors such as Epstein–Barr virus (EBV) have also been associated with clinical phenotypes and p53 expression changes in OLP, suggesting possible involvement in epithelial dysregulation [5]. In addition to these established factors, viral infections, particularly human herpesviruses, have long been proposed as potential contributors to disease initiation, exacerbation, and persistence [6,7,8].

EBV, a ubiquitous gamma herpesvirus infecting over 90% of adults worldwide, establishes lifelong latency in B lymphocytes and oral epithelial cells. Key latency-associated proteins, including LMP1, EBNA family proteins, and BARF1, can modulate immune signaling, epithelial proliferation, and chronic inflammatory pathways [9,10]. Several studies have reported on the presence of EBV DNA or antigens in OLP lesions, suggesting that EBV may amplify immune-mediated epithelial injury, alter basal keratinocyte antigenicity, or enhance local inflammatory responses [11]. However, other investigations have failed to demonstrate a significant EBV association with this disorder, and the virus’s role in OLP etiopathogenesis remains controversial.

Human herpesvirus-7 (HHV-7), a β-herpesvirus with marked tropism for CD4+ T lymphocytes, establishes lifelong persistency after primary infection, is frequently shed in saliva, and demonstrates efficient transmission within human populations, contributing to its widespread prevalence [12,13]. HHV-7 encodes genes such as U12, ORF50, and ORF73, which facilitate reactivation and immune evasion. Detection of HHV-7 DNA in oral tissues and saliva of patients with recurrent aphthous ulcers has led to speculation that HHV-7 may act as a viral trigger through direct cytopathic effects, induction of T-cell dysregulation, or post-viral inflammatory activation [14]. Nonetheless, as with EBV, findings across studies remain inconsistent.

OLP itself is characterized by a T-cell-mediated autoimmune attack on basal keratinocytes, with classical histopathological findings of a band-like lymphocytic infiltrate, basal cell degeneration, and chronic epithelial inflammation [11]. Viral triggers capable of modifying epithelial antigens, activating latent immune pathways, or sustaining chronic inflammation could plausibly influence OLP pathophysiology [15]. On the contrary, AS is believed to result from a combination of mucosal barrier fragility, local trauma, systemic predisposition, stress, and immune dysregulation, with viral reactivation considered a potential contributing factor rather than a primary cause [4].

Given the inconsistent evidence and the high prevalence of herpesviruses even in healthy populations, there is a need for well-designed studies that directly compare salivary viral loads and levels of virus-specific antibodies across clinically defined groups and healthy controls. Therefore, this study investigates the presence of EBV and HHV-7 biomarkers in saliva and serum of patients with OLP, AS, and healthy controls. Our aim was to determine whether herpesvirus activity correlates with disease presence or severity when adjusting for demographic and clinical confounders, thereby clarifying their potential contributory role in the pathogenesis of OLP and AS.

## 2. Materials and Methods

### 2.1. Study Group

Study design and population: This comparative observational study was conducted at the Riga Stradiņš University (RSU) Institute of Stomatology, Centre of Oral Medicine, Riga, Latvia, between June 2021 and August 2024. The primary aim was to investigate the possible role of EBV and HHV-7 in the pathogenesis of OLP and AS by analyzing salivary viral loads and the level of virus-specific antibodies in serum.

A total of 100 adult participants aged 18–75 years were enrolled in the study following written informed consent and ethical approval from the RSU Ethics Committee. Participants were assigned to three groups based on clinical diagnosis: OLP group (n = 35) patients with clinically and histopathologically confirmed diagnosis, including reticular, papular, plaque-like, and erosive forms; AS group (n = 31) patients with recurrent aphthous stomatitis diagnosed clinically (minor, major, or herpetiform ulcerations), and control group (n = 34) individuals without clinical history or signs of OLP, AS, or other oral mucosal disease.

Demographic data, medical history, allergies, stress-related information, and discomfort levels were recorded for all participants.

Inclusion criteria included adults aged 18–75 years who provided written informed consent, had a confirmed diagnosis of OLP (clinical and histopathological) or AS (clinical), and were able to provide saliva samples, relevant medical information, and complete the questionnaires.

Exclusion criteria included participants younger than 18 years or older than 75 years, oral mucosal diseases other than OLP or AS (e.g., traumatic, infectious, autoimmune ulcerations), and persons who were unable or unwilling to provide mandatory saliva samples or to sign informed consent.

### 2.2. Sample Collection

Saliva collection: About 2 mL of unstimulated whole saliva was collected using Oragene OG-500 kits (DNA-Genotek, Ottawa, ON, Canada) following a standardized sterile technique and mixed with the stabilizing solution, resulting in a total volume of approximately 3 mL. Samples were stored at −80 °C and transported to the RSU Institute of Microbiology and Virology for molecular analysis.

Serum collection: Venous blood samples were collected in 5 mL BD Vacutainer^®^ serum tubes, yielding approximately 2.2–2.8 mL of serum per patient for the assessment of EBV-specific IgG and IgM levels, and were processed according to standard protocols in an accredited clinical laboratory (E.Gulbis Laboratory). Serum was aliquoted, frozen, stored, and transported to the RSU Institute of Microbiology and Virology for subsequent evaluation of HHV-7-specific IgG and IgM serostatus using optical density-based ELISA.

#### 2.2.1. Laboratory Analysis

DNA Extraction and Real-Time PCR Quantification:

Salivary DNA was isolated from Oragene^®^ OG-500 collection tubes using the prepIT-L2P reagent (DNA Genotek, Ottawa, ON, Canada) with 500 µL of the sample in accordance with the manufacturer’s protocol. Quantification of herpesvirus DNA was performed using commercially available real-time PCR assays. EBV DNA load was determined using the EBV Real-TM Quant kit (Sacace Biotechnologies, Como, Italy), with a linear quantification range of 500 to 1 × 10^7^ copies/mL; values below this limit were reported as <500 copies/mL. HHV-7 DNA load was measured using the REALQUALITY RQ-HHV-7 kit together with the corresponding quantification standards (AB Analitica, Padua, Italy), with a linear quantification range corresponding to 250 to 5 × 10^8^ copies/mL, depending on extraction parameters. Viral loads were expressed as copies per milliliter of saliva, and only values within the manufacturer-defined linear ranges were considered quantitatively reliable.

#### 2.2.2. Serological Analysis

EBV and HHV-7 specific antibodies in blood sera were assessed using ELISA-based methods in accordance with the manufacturers’ instructions. EBV-specific IgG and IgM antibodies were detected using automated chemiluminescent immunoassays (LIAISON^®^ EBV VCA IgG and LIAISON^®^ EBV IgM; DiaSorin S.p.A., Saluggia, Italy), using 10 µL of serum per reaction with results interpreted according to kit-defined cut-off values.

HHV-7 specific IgG and IgM antibodies were analyzed using qualitative ELISA kits (BT LAB, Jiaxing, China) (10 µL of serum diluted with 40 µL of sample diluent per reaction) with optical density measured by a spectrophotometer at 450 nm. Samples were classified as positive or negative based on the manufacturer’s interpretation criteria, allowing assessment of prior exposure and/or recent immune activation rather than quantitative serological measurement.

### 2.3. Statistical Data Analysis

All data were initially characterized using descriptive statistics. Continuous variables were presented as mean and standard deviation (SD) or median and interquartile range (Q1–Q3), as determined by their distribution. Categorical variables were presented as counts and percentages.

Initial examination of the key continuous variables via Shapiro–Wilk tests and visual inspection of Q-Q plots revealed significant deviations from normality. This non-normal distribution was accounted for in subsequent inferential testing, primarily through the use of non-parametric methods and bootstrapping.

To identify baseline differences in continuous and categorical characteristics between the research groups, a univariate analysis was performed. Differences in continuous non-normally distributed variables (including viral loads and antibody concentrations) across the three research groups were assessed using the Kruskal–Wallis H test. Differences in proportions for categorical variables (e.g., gender and discomfort level) between the groups were evaluated using the Chi-square test of homogeneity. Fisher’s exact test was utilized when the expected frequencies in any cell of the contingency table were less than five. To investigate the preliminary relationships between continuous variables (viral loads, antibody titers, and age), Spearman’s rank correlation test was run.

To evaluate the association between the study groups (OLP, AS, and Control) and the primary outcomes (viral loads, optical density, and immunoglobulin concentrations), multivariable regression models were employed. Given the non-negative and right-skewed distribution of the biological markers, Generalized Linear Models (GLM) with a Gamma distribution and a log-link function were utilized for viral load (HHV-7 copies/mL saliva), optical density (OD_IgG and OD_IgM), and immunoglobulin concentration (EBV IgG IU/mL and IgM IU/mL). The EBV copies/mL saliva data exhibited a high proportion of zero-value observations (33%), rendering standard Gamma models unsuitable. Consequently, a Hurdle model was implemented.

In all models, age, gender, discomfort level, treatment modality, and the presence of chronic diseases were included as covariates to control for potential confounding influences. Results for the GLM and the intensity component of the Hurdle model are reported as Adjusted Mean Ratios (AMR) or Fold Changes (FC), representing the exponentiated regression coefficients.

The results were considered statistically significant when the *p*-value was <0.05. Viral load data (copies/mL) were non-normally distributed and were therefore log10-transformed for visualization. For statistical comparisons between clinical forms, the non-parametric Mann–Whitney U test was employed.

For statistical analyses, clinical forms of OLP were categorized into two groups: aggressive forms (erosive and atrophic), representing the highest levels of symptoms and discomfort, and non-aggressive forms (reticular and plaque-like). For AS, comparisons were performed between minor and herpetiform types, as no participants presented with major aphthous ulcers.

Statistical data analysis was performed with Jamovi (v.2.7.11).

## 3. Results

Salivary EBV and HHV-7 loads varied considerably among participants across all study groups, ranging from undetectable to high copy numbers. In the OLP group, EBV DNA loads demonstrated wide variability, spanning approximately 10 to 10 × 10^7^ copies/mL, while HHV-7 DNA loads ranged from about 2.5 × 10^2^ to 2.5 × 10^6^ copies/mL. In the AS group, HHV-7 loads were observed within a similar range (approximately 2.4 × 10^3^ to 2.4 × 10^6^ copies/mL), whereas EBV DNA loads varied from undetectable to around 10 × 10^5^ copies/mL. In the control group, HHV-7 DNA loads ranged from 1.8 × 10^3^ to 5.2 × 10^6^ copies/mL, and EBV DNA loads from 1.36 to 2.5 × 10^7^ copies/mL, indicating baseline herpesvirus shedding in individuals without clinically evident oral inflammatory disease.

**Figure 1:**
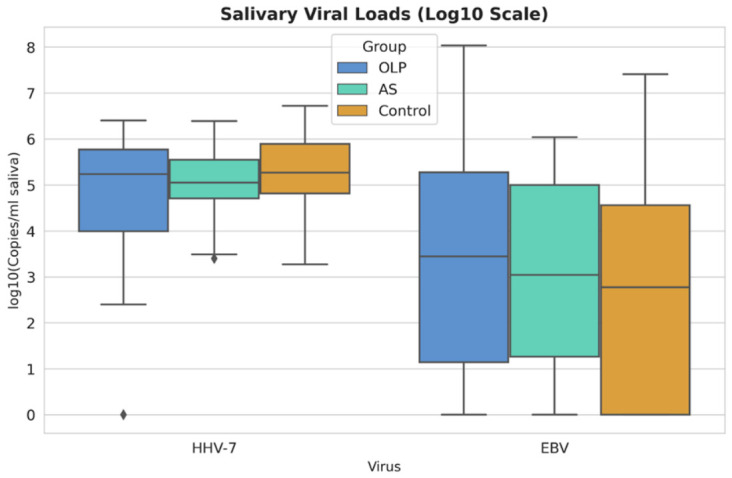
Salivary HHV-7 and EBV loads (log10 copies/mL saliva) in patients with OLP, AS, and healthy controls. Box plots represent median values, interquartile ranges, and distribution of viral loads across study groups. The diamond indicates an outlier.

Figure 1 shows salivary HHV-7 and EBV loads across OLP, AS, and control groups on a log10 scale. HHV-7 loads were comparable across all groups, whereas EBV demonstrated greater variability, particularly in OLP, with a wider range of viral loads.

**Figure 2:**
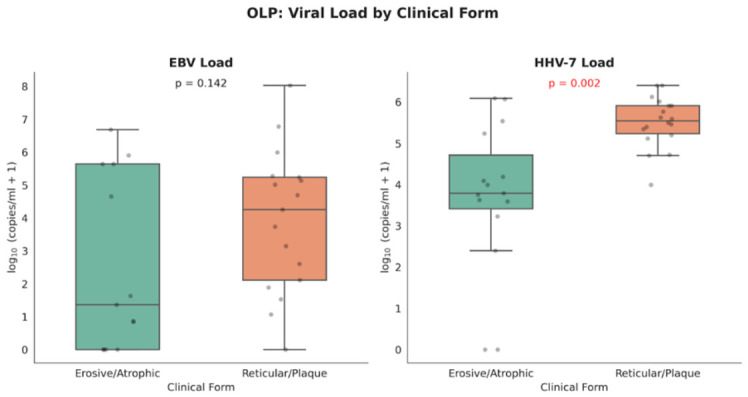
Comparison of salivary EBV and HHV-7 loads (log10 copies/mL saliva) between aggressive (erosive/atrophic) and non-aggressive (reticular/plaque) clinical forms of OLP. Box plots represent median values, interquartile ranges, and distribution of individual measurements. Dots represent individual data point.

Figure 2 illustrates the comparison of EBV and HHV-7 salivary loads between aggressive (erosive/atrophic) and non-aggressive (reticular/plaque) clinical forms of OLP. HHV-7 load differed significantly between clinical forms (Mann–Whitney U = 52.0, *p* = 0.002), with higher median levels observed in patients with the reticular/plaque form (median 3.5 × 10^5^ copies/mL) compared with those with the erosive/atrophic form (median 6.1 × 10^3^ copies/mL), suggesting a potential association between increased HHV-7 infection and non-aggressive OLP presentations. In contrast, EBV load showed a similar trend but did not reach statistical significance (*p* = 0.142).

**Figure 3:**
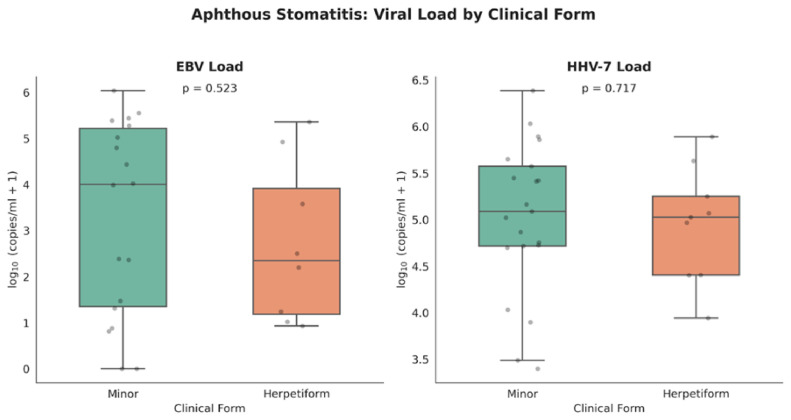
Comparison of salivary EBV and HHV-7 loads (log10 copies/mL saliva) between minor and herpetiform clinical forms of AS. Box plots represent median values, interquartile ranges, and distribution of individual measurements. Dots represent individual data point.

Figure 3 illustrates the comparison of EBV and HHV-7 loads between minor and herpetiform clinical forms of AS. No statistically significant differences in viral load were observed between the two clinical forms for either EBV (*p* = 0.523) or HHV-7 (*p* = 0.717). Although minor aphthous stomatitis demonstrated slightly higher variability in viral load distribution, overall viral loads were comparable between groups.

### 3.1. Baseline Characteristics and Univariate Comparison

The baseline characteristics and univariate comparisons between the research groups are summarized in Table 1. The analysis revealed statistically significant differences across the groups in three key areas: age, EBV IgG level, and discomfort level.

There was a highly significant difference in the mean age of participants across the groups (*p* < 0.001). Specifically, the OLP group was considerably older than both the AS and control groups. This finding strongly supports the inclusion of age as a confounding covariate in the subsequent multivariate analysis to control for this difference in the comparison of groups.

The Kruskal–Wallis H test demonstrated a statistically significant difference in EBV IgG levels across the three groups (*p* = 0.016). The highest median level was observed in the OLP group (Median: 636 IU/mL), followed by the control group (Median: 293 IU/mL), and the lowest in the AS group (Median: 155 IU/mL).

The patient-reported discomfort level showed a highly significant difference across the groups (*p* < 0.001), which is expected given the clinical diagnoses. The control group reported 100% “none,” while the AS group reported the highest rate of “severe” discomfort (67.7%). The OLP group exhibited a more distributed severity pattern.

**Figure 4:**
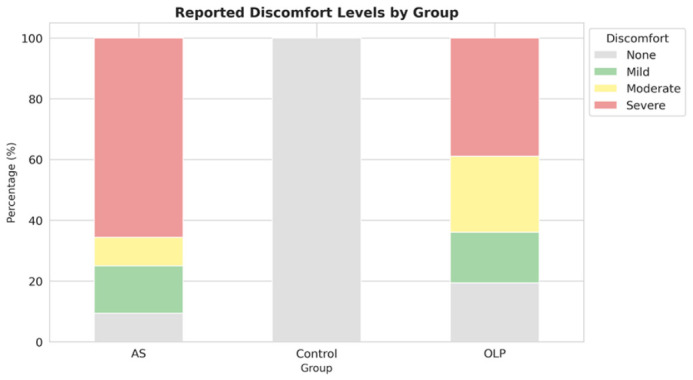
Distribution of self-reported discomfort levels among participants with AS, OLP, and healthy controls. Bars represent the percentage of participants reporting no discomfort, mild, moderate, or severe symptoms within each group.

As illustrated in Figure 4, reported discomfort levels varied among participants with AS, OLP, and healthy controls. The control group reported no discomfort (100%), whereas both the AS and OLP groups demonstrated varying degrees of symptoms. Severe discomfort predominated in the AS group, while the OLP group showed a more balanced distribution across mild, moderate, and severe discomfort levels, indicating a substantial symptomatic burden in both disease groups compared to controls.

In contrast, no statistically significant differences (*p* > 0.05) were found across the groups for viral loads, levels of virus-specific antibodies in serum, and gender.

### 3.2. Correlation Analysis

The strongest correlation was observed between age and EBV IgG IU/mL, showing a moderate positive association (r = 0.426, n = 99, *p* < 0.001). This suggests that higher levels of EBV IgG antibodies are significantly associated with older age in the study population.

Age demonstrated significant weak negative correlations with HHV-7 copies/mL saliva (r = −0.242, n = 99, *p* = 0.016) and weak positive correlations with EBV load copies/mL saliva (r = 0.206, n = 99, *p* = 0.041).

A moderate positive correlation was identified between the raw optical density readings for HHV-7 antibodies, OD_IgG and OD_IgM, (r = 0.412, n = 63, *p* < 0.001). Age also showed a weak negative correlation with the raw optical density reading, OD_IgG (r = −0.290, n = 65, *p* = 0.019).

**Figure 5:**
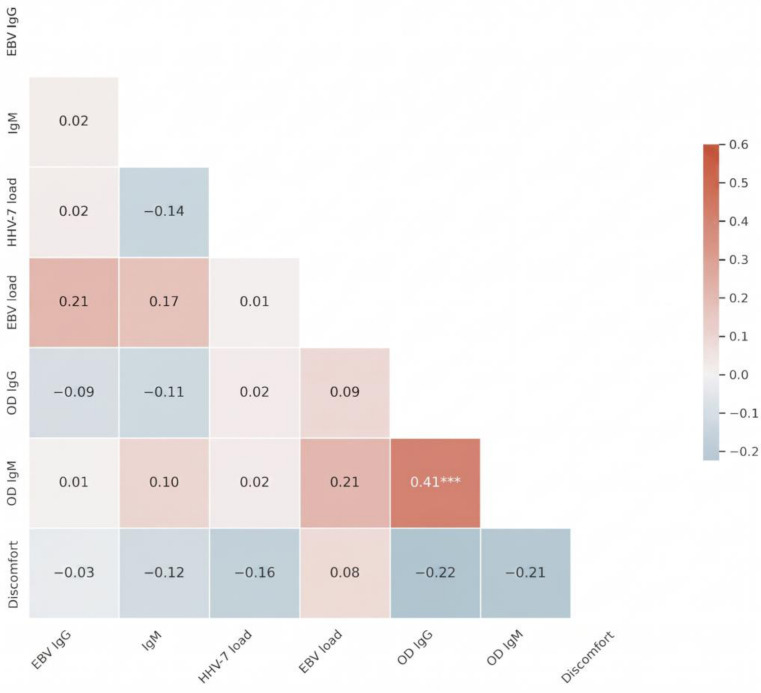
Heatmap of Spearman correlation coefficients among virological, immunological, and clinical markers. Statistical significance is indicated by asterisks (*** *p* < 0.001).

Spearman rank correlation analysis revealed a highly significant positive association between HHV-7 specific OD IgG and OD IgM levels (r = 0.410, *p* < 0.001), confirming the internal consistency of the serological data. In contrast, no significant correlation was observed between salivary EBV and HHV-7 DNA loads (r = 0.010, *p* = 0.913), indicating that the shedding patterns of these two viruses are independent in the study population. Regarding clinical associations, self-reported discomfort levels showed weak, non-significant correlations with salivary viral loads and EBV-specific IgG levels (Figure 5).

### 3.3. Multivariable Regression Results

After adjusting for age, gender, discomfort level, treatment, and chronic diseases in the multivariable models, the diagnostic group exhibited a selective impact on the analyzed outcomes. Diagnostic group was not independently associated with HHV-7 salivary load (χ^2^(2) = 3.89, *p* = 0.143), EBV salivary load (χ^2^(2) = 0.01, *p* = 0.992), OD_IgG (χ^2^(2) = 4.86, *p* = 0.088), OD_IgM (χ^2^(2) = 3.61, *p* = 0.164), or EBV IgG levels (χ^2^(2) = 0.30, *p* = 0.859). In contrast, the diagnostic group was a significant independent predictor of IgM levels (χ^2^(2) = 12.60, *p* = 0.002), primarily driven by significantly elevated concentrations in the AS group compared to controls (AMR = 3.487, *p* < 0.001) (Table 2).

## 4. Discussion

In this comparative observational study, the predominance of female participants may reflect reporting patterns observed in previous studies [16,17,18,19], where females are more frequently represented; however, it remains unclear whether this reflects true disease prevalence or differences in healthcare-seeking behavior, and potential selection bias cannot be excluded. The marked age difference between the OLP [20,21] and control groups represents a potential confounding factor that may have influenced the serological findings through age-related effects. In contrast, the more comparable age distribution between the AS and control groups suggests that observations related to AS [22] may be more reliable within the context of this study. These differences should be taken into account when interpreting the results.

In this cross-sectional study, both EBV and HHV-7 genomic sequences were widely detected in saliva across patients with OLP, AS, and healthy controls, underscoring their high baseline prevalence and frequent oral shedding, consistent with earlier reports [23,24]. Although EBV IgG levels appeared higher in OLP in univariate testing, these differences disappeared once age was included in the multivariate models, indicating that age-related immunological memory rather than disease status was the principal determinant of EBV seropositivity. This finding aligns with the well-established pattern of age-dependent increases in EBV IgG levels [25,26]. Together, these results suggest that EBV and HHV-7 may play contributory but non-specific roles in oral mucosal inflammation rather than acting as primary etiological drivers of OLP or AS [27,28]. The novelty of this study lies in the combined evaluation of salivary EBV and HHV-7 loads together with serological markers across clinically defined groups, with adjustment for key confounders such as age and comorbidities, allowing a more precise assessment of their independent relevance.

The potential involvement of EBV in OLP has been widely investigated, with reported detection rates varying according to sample type, geographic population, and diagnostic methodology. A systematic review and meta-analysis including observational studies with histopathologically confirmed OLP cases demonstrated a significant association between EBV infection and OLP, with a pooled odds ratio of 4.41 (95% CI: 2.74–7.11) [29]. Beyond epidemiological associations, recent evidence suggests possible mechanistic pathways linking EBV to OLP pathogenesis, including chronic inflammatory activation and pro-oncogenic signaling processes. Additionally, in erosive OLP, EBV-infected plasma cells have been observed more frequently [30]. Mechanistically, EBV latency proteins such as LMP1, EBNA1, and BARF1 have the capacity to enhance epithelial inflammation, alter antigen presentation, and amplify CD8^+^ T-cell infiltration, theoretically contributing to the immunopathological processes characteristic of OLP [31]. However, the literature data remain inconsistent. Some investigations have failed to detect EBV in OLP lesions altogether, highlighting population heterogeneity, variations in sampling depth, and differences in the phases of viral latency or reactivation [32]. In our study, salivary EBV DNA loads did not differ significantly between diagnostic groups, supporting the concept that EBV may influence disease only in specific subgroups or under conditions that promote epithelial vulnerability or immune dysregulation rather than serving as a universal pathogenic factor.

HHV-7 showed similar patterns. Although frequently detected in saliva and adenoid tissues of healthy individuals and widely recognized as a persistent oral resident with detection rates as high as 40–80% [33], HHV-7′s specific contribution to OLP or AS remains poorly defined. While some studies have reported HHV-7 in recurrent aphthous ulcers or adenotonsillar disease [28], our results did not demonstrate any group-specific differences in salivary HHV-7 load. This reinforces the idea that HHV-7 represents a background viral signal of the oral ecosystem, with any clinical relevance likely to be secondary, indirect, or dependent on external triggers such as stress, immune fluctuations, or co-infections [34]. The results of the present study do not allow firm conclusions to be drawn regarding the mechanisms through which herpesviruses may contribute to oral diseases, such as modulation of antigen-presenting cells and T-cell activation [35]. Moreover, the cross-sectional design of the study precludes the establishment of causal relationships; therefore, the observed associations between viral markers and clinical conditions should be interpreted as associative rather than causal. Therefore, further investigations are warranted, particularly in OLP cases with higher viral loads, including longitudinal studies and immunohistochemical evaluation of biopsy specimens to better elucidate the role of herpesviruses in disease pathogenesis.

The discrepancies between our findings and studies that previously reported stronger viruses and disease associations can be attributed to several factors, including differences in detection methods (biopsy vs. saliva), limited sample sizes, variations in OLP subtypes, and the universal nature of herpesvirus latency in epithelial and lymphoid tissues. Studies focusing specifically on erosive OLP may have observed stronger associations simply because these lesions exhibit greater inflammatory activity, more epithelial disruption, and potentially more viral reactivation. Furthermore, the ubiquity of herpesviruses in healthy populations complicates causal interpretation and underscores the need for careful adjustment for confounding variables such as age [36].

This study has limitations that should be considered when interpreting the findings. Although saliva sampling is non-invasive and clinically practical, it may not fully reflect tissue-level latent infection. In addition, serum and biopsy samples were not uniformly available across all study groups, which may have limited the consistency of cross-sample comparisons. The serological assessment in the present study utilized EBV VCA IgG and IgM, which are widely accepted as standard markers for determining infection status in both clinical and research settings; similar approaches have been applied in previous studies investigating EBV in oral mucosal diseases, supporting the methodological validity of the present analysis [25,37,38]. Nevertheless, the use of a limited serological panel may not fully capture all aspects of viral latency and reactivation.

In the OLP group, chronic conditions such as hypertension, GERD, hypercholesterolemia, cardiac disease, diabetes, hypothyroidism, autoimmune thyroiditis, hepatitis C, and antidepressant use were frequently reported, often coexisting within the same individuals rather than occurring as isolated factors, consistent with previous studies [39,40,41,42,43]. A similar pattern was observed in the AS group, where patients commonly presented with multiple comorbidities, including gastrointestinal, cardiovascular, endocrine, metabolic, infectious, autoimmune, renal, and psychological conditions [44,45,46]. Across both groups, these comorbidities showed no consistent association with clinical severity, salivary viral load, or serological antibody levels, suggesting a non-specific or multifactorial role rather than acting as independent determinants [42,43,44,45,46,47]. Nevertheless, patients with a greater comorbidity burden or immune dysregulation may be more susceptible to viral reactivation and disease exacerbation, although this could not be evaluated in the present study. The cross-sectional nature of the study further limits the ability to assess temporal relationships between viral activity and disease flares. In addition, age imbalance across groups, particularly with older participants in the OLP cohort, may have influenced serological patterns despite statistical adjustment. Additionally, we did not evaluate other biomarkers such as cytokines, cortisol, or co-infecting viruses, which could interact with herpesvirus reactivation or mucosal immunity.

Nevertheless, the uniform detection of both EBV and HHV-7 across all study groups, together with the lack of independent associations after multivariable adjustment, supports the view that OLP and AS are multifactorial conditions. Their pathogenesis likely reflects a complex interaction among host immune responses, epithelial barrier integrity, psychological stress, genetic susceptibility, and microbial or viral exposures. In this framework, herpesviruses are more likely to function as secondary, context-dependent modifiers rather than as consistent biomarkers or primary causative agents.

A formal prior sample size calculation was not performed because reliable estimates of expected effect sizes for salivary EBV and HHV-7 biomarkers in OLP and AS were not available at the design stage. An exploratory post hoc power assessment suggested that the present sample size was sufficient to detect moderate-to-large between-group effects (power = 0.88), but may have been underpowered for small effects, particularly for outcomes with substantial interindividual variability. Therefore, negative findings should be interpreted cautiously.

## 5. Conclusions

This study demonstrates that salivary EBV and HHV-7 DNA are consistently detectable across patients with OLP, AS, and healthy controls, indicating a high baseline level of viral shedding independent of disease status. After adjustment for age, gender, and clinical covariates, neither EBV nor HHV-7 salivary loads showed independent associations with OLP or AS, and HHV-7 distribution remained uniform across all groups. Although EBV IgG levels were initially higher in OLP, this difference was attributable to age-related effects rather than disease-specific immune activity.

The absence of significant associations in multivariable models, together with the high interindividual variability observed in EBV and HHV-7 salivary loads, suggests that these viruses do not serve as reliable standalone biomarkers for distinguishing inflammatory oral mucosal diseases from healthy states. The only disease-related signal identified was an increase in IgM levels in the AS group, which may reflect transient immune activation rather than a direct viral effect.

Overall, the findings support a model in which EBV and HHV-7 function as potential cofactors that may modulate disease processes under specific conditions, with their relevance confined to particular clinical contexts rather than reflecting a consistent independent etiological role. Future studies should incorporate longitudinal sampling, lesion-specific tissue analysis, and integrated immunological profiling to clarify whether viral activity contributes to disease dynamics in defined patient subsets.

## Figures and Tables

**Table 1:** Baseline demographic, clinical, and immunological characteristics of the research groups.

Parameter	AS	Control	OLP	*p*	Effect Size
EBV IgG, median (Q1–Q3)	155 (46–738) ^a^	293 (141–622) ^b^	636 (240–750) ^ab^	0.016	0.109
IgM, median (Q1–Q3)	7 (5–12)	9 (6–10)	6 (4–10)	0.327	NA
HHV-7 copies/mL, median (Q1–Q3)	112,348 (50,537–353,024)	170,117 (59,201–758,755)	157,997 (5937–508,514)	0.361	NA
EBV copies/mL, median (Q1–Q3)	231 (6–73,936)	1 (0–5490)	129 (0–156,028)	0.171	NA
OD_IgG, median (Q1–Q3)	0.045 (0.042–0.049)	0.05 (0.043–0.06	0.046 (0.042–0.05)	0.337	NA
OD_IgM, median (Q1–Q3)	0.046 (0.043–0.052)	0.047 (0.043–0.053)	0.044 (0.042–0.053)	0.564	NA
Age, mean (SD)	38.7 (13.6)	36.1 (14.7)	58.5 (10.9)	<0.001	0.391
Gender, n (%)				0.501	NA
Female	20 (64.5)	23 (67.6)	27 (77.1)		
Male	11 (35.5)	11 (32.4)	8 (22.9)		
Discomfort level, n (%) *				<0.001	NA
None	2 (6.5)	34 (100)	7 (20.0)		
Mild	5 (16.1)	0	6 (17.1)		
Moderate	3 (9.7)	0	9 (25.7)		
Severe	21 (67.7)	0	13 (37.1)		

Note: ^a^, ^b^ values with different superscript letters in the same row indicate significant differences (*p* < 0.05); Q1–Q3–the first and third quartiles; NA—not applicable; HHV 7—Human herpes virus 7; EBV—Epstein–Barr Virus. * indicate difference between AS and OLP groups.

**Table 2:** Multivariable regression analysis of viral loads and immunological markers adjusted for clinical and demographic covariates.

Outcome	Parameter (vs. Control)	AMR/FC	95% CI
HHV-7 viral load	OLP	1.138	[0.160, 8.113]
	AS	0.395	[0.137, 1.143]
EBL viral load	OLP	7.95	[0.21, 298.4]
	AS	2.42	[0.03, 184.2]
OD_IgG	OLP	0.139	[0.018, 1.052]
	AS	0.515	[0.229, 1.155]
OD_IgM	OLP	0.155	[0.019, 1.282]
	AS	0.574	[0.247, 1.333]
EBV IgG (IU/mL)	OLP	1.579	[0.503, 4.961]
	AS	0.523	[0.304, 0.901]
IgM (IU/mL)	OLP	2.901	[0.620, 13.583]
	AS	3.487	[1.675, 7.261]

Note: AMR (Adjusted Mean Ratio) and FC (Fold Change) represent the exponentiated regression coefficient. All models were adjusted for age, gender, discomfort level, treatment, and chronic diseases.

## Data Availability

The data supporting the findings of this study are included within the article. Additional anonymized data may be made available by the corresponding author upon reasonable request for non-commercial research and educational purposes, subject to ethical approval and institutional regulations.
